# Effect of prolactin on normal and keratoconus human corneal stromal fibroblasts *in vitro*

**DOI:** 10.1371/journal.pone.0249344

**Published:** 2021-04-01

**Authors:** Philipp Anders, Xuefei Song, Bence György, Nora Szentmary, Berthold Seitz, Zisis Gatzioufas

**Affiliations:** 1 Department of Ophthalmology, University Hospital Basel, Basel, Switzerland; 2 Institute of Molecular and Clinical Ophthalmology Basel, Basel, Switzerland; 3 Department of Ophthalmology, Shanghai Ninth People’s Hospital, Shanghai, China; 4 Department of Ophthalmology, Saarland University Medical Center, Homburg/Saar, Germany; 5 Dr. Rolf M. Schwiete Center for Limbal Stem Cell Deficiency and Congenital Aniridia Research, Saarland University, Homburg/Saar, Germany; Cedars-Sinai Medical Center, UNITED STATES

## Abstract

**Purpose:**

To examine the effect of prolactin (PRL) on human corneal stromal fibroblasts (CSFs), derived from healthy individuals and from keratoconus (KC) patients, *in vitro*, specifically assessing physiological and elevated PRL concentrations as apparent during pregnancy.

**Methods:**

Eye bank corneas of 3 female and 3 male healthy individuals as well as the corneal buttons of 3 female and 3 male KC patients were utilized for this study. The endothelium of the cornea was removed with sterile surgical scalpels, the probes were washed repeatedly with Dulbecco’s PBS and corneoscleral rims were trimmed off. Subsequently the corneal stroma was digested with collagenase type I and the harvested CSFs were cultured. We then examined (1) cell proliferation, (2) cell viability and (3) cytokine release of CSFs upon exposure to prolactin *in vitro*.

**Results:**

With respect to viability and proliferation our experiments did not show significant differences between CSFs exposed to different PRL concentrations. Our data show a significantly lower IL-8 concentration in normal CSFs exposed to 10ng/ml PRL compared to 0ng/ml and 1000ng/ml at 5 hours post exposition. Moreover, we can report significantly lower secretion of IL-8, IL-6, HGF, VEGF and FGFb in KC CSFs compared to normal CSFs, independent of PRL exposure, as determined by cytokine ELISA.

**Conclusion:**

Our data in part points towards corneal cytokine secretion as a possible link between altered stromal PRL concentrations and KC progression. However, in our small dataset a significant influence of PRL concentration on cytokine secretion can only be described for IL-8 in normal CSFs. Further our results contribute to existing reports on the importance of cytokines in KC development, with an emphasis on significantly lower cytokine secretion in KC CSFs compared to normal controls.

## Introduction

Keratoconus (KC) is a progressive condition, in which the cornea acquires an ectatic cone-like shape, which is caused by thinning of the corneal stroma [1]. In developed countries, KC is the most frequent corneal ectatic disorder with a prevalence of 54.5 per 100’000 [2]. The underlying pathophysiological mechanisms of KC are not yet fully elucidated. Even though KC has been described as a non-inflammatory disease for a long time, recent studies have reported a significant role of cytokines in KC development, suggesting the involvement of inflammatory mechanisms [3, 4]. On the cellular level, keratocytes are the housekeepers of the corneal stroma, contributing to corneal homeostasis [5]. Normally quiescent, these cells are activated in pathological processes and, therefore, hold a pivotal position in the KC trajectory [6]. Additionally, hormonal imbalances have been connected to KC development: for instance, changes in T4 concentrations, like apparent in thyroid gland dysfunction, have been shown to alter the expression of collagens within the cornea [7].

Pronounced hormonal changes occur during pregnancy and reports on the development of corneal ectasia during pregnancy [8] or upon treatment with estrogenic activity regulator [9], sparked an increased interest into the effect of pregnancy associated hormones on corneal ectasia. Prolactin (PRL) is a pituitary hormone, which is progressively expressed towards the end of pregnancy, and which is commonly known for its function of stimulating milk production in the female mammary gland [10]. However, apart from this function, PRL has been reported to exert more functions than all other pituitary hormones combined. PRL Receptors (PRLRs) are not only expressed in the mammary gland but also in the male testis, the central nervous system, the kidney and many other organs. Accordingly, PRL functions comprise contributions to the water and electrolyte balance, to social behavioral patterns and various others [11].

With respect to KC it is of particular interest that PRL has been reported to increase both cytokine and cytokine receptor expression [11]. As cytokines have lately been attributed a significant role in KC development [3, 12, 13], our here presented study investigates the effect of PRL on cultured human corneal keratocytes, specifically assessing cytokine expression. Corneal keratocytes become corneal stromal fibroblasts (CSFs) when cultured and will therefore be termed CSFs from here on.

It has been shown previously that the eye’s aqueous humor contains PRL [14]. Further the corneal endothelium is permeable to macromolecules > 150kDa [15], which would allow altered PRL concentrations in the aqueous humor to exert an effect on corneal keratocytes. In this study we exposed CSFs to PRL concentrations of 0, 0.1, 1, 10, 100 and 1000 ng/ml, which reflect normal and pregnancy PRL blood concentrations [16], as well as PRL concentrations measured in aqueous humor [14], since no measurements of PRL concentrations within the corneal stroma are available. To our knowledge no publications are available on the effect of PRL on the cornea. Nevertheless PRL has been described to influence collagen organization in 3D tissue culture models [17]. Even though KC is reported to be more frequent in males, it has been stated, that gender does not affect the trajectory of the disease [18]. Therefore we combine male and female CSF subgroups in certain analyses to increase statistical validity. The purpose of this study is, to examine the effect of prolactin on CSFs, *in vitro*, specifically assessing physiological and elevated prolactin concentrations as apparent during pregnancy.

## Materials and methods

The study was performed in agreement with the tenets of the declaration of Helsinki and the ARVO statement for the use of human tissues.

This study was conducted in compliance with and approved by the local human ethics committee (IRB) of Saarland University, Homburg/Saar, Germany. Written informed consent was obtained from all subjects for the use of their corneal buttons for this study. Eyebank corneas were procured from the LIONS-Hornhautbank Saar-Lor-Lux, Trier/Westpfalz.

### Materials

Dulbecco’s Modified Eagle Medium: (Nutrient Mixture F-12 (DMEM/F12)); fetal bovine serum (10%); P/S (1% of 10,000U/ml penicillin and 10mg/ml streptomycin); 0.05% trypsin; 0.02% ethylenediaminetetraacetic acid (EDTA) were purchased from PAA Laboratories (Pasching, Austria), Collagenase A, Dispase II were obtained from Roche Diagnostics (Mannheim, Germany). All tissue culture plastics were from BD Biosciences (Heidelberg, Germany). Prolactin (682-PL) was purchased from R&D Systems (Wiesbaden, Germany).

### Keratocyte isolation and cell cultures

Normal and KC corneas were matched for sex and age (Ages of corneal donors: 43, 32, 30, 38, 27, 33; Ages of KC patients: 42, 31, 29, 36, 26, 32). Donor corneas had not been subjected to previous surgeries like corneal cross-linking. After removing the corneal endothelium with sterile surgical disposable scalpels, the Eye Bank corneas (three male and three female) and the keratoconus corneal buttons (three male and three female) were washed repeatedly with Dulbecco’s PBS without Ca^2+^ and Mg^2+^ (PAA, Pasching, Austria). The corneoscleral rims were trimmed off and a corneal button was cut by a 8.0 mm diameter trephine. Following this initial preparation, corneal buttons were moved to a 24-well culture plate with 1ml Dispase II (Roche, Mannheim, Germany) (2.4U/ml in medium). After incubation with Dispase in a humidified 37°C incubator with 5% CO_2_ for 4 hours, the epithelium of the cornea was removed with a sterile surgical disposable scalpel.

The stroma was washed for three times with PBS and medium and then totally digested with 1mg/ml collagenase type I (Sigma, Deisenhofen, Germany). Digestion was carried out at 37°C for 10 hours after which the cells were washed with medium containing 5% fetal bovine serum (FBS) (PAA, Pasching, Austria). The particulate material from each of our twelve study corneas, were individually seeded into one well of a 6 well culture plate containing 2ml of medium.

Cells were grown in DEME/F-12 HAM (Sigma, Deisenhofen, Germany) supplemented with 10% FBS, 1% penicillin (100U/ml)/ streptomycin (100μg/ml) (PAA, Pasching, Austria) at 37°C in a 5% CO_2_/air incubator and passaged by standard trypsinisation using 1× trypsin (2.5 M)/EDTA (0.38M) solution (PAA, Pasching, Austria). After confluence was reached in a 75cm^2^ flask, the cultures were split 1:3. Further, for all experiments in this study cells were seeded in 96 well culture plates at 4,000 cells/well. All experiments were conducted after the third passage and cells were at similar confluence. Corneal keratocytes became corneal stromal fibroblasts (CSFs) when cultured and had fibroblastic appearance.

### BrdU proliferation assay

Cell Proliferation (CP) of CSFs was determined *in vitro* using a BrdU proliferation ELISA kit (Roche, Mannheim, Germany), which detects BrdU incorporation into newly synthesized DNA of actively proliferating cells. The assay was conducted prior to and after 24 hours of hormone treatment according to the manufacturer’s instructions. Briefly, 10μl of BrdU labeling solution were added to each well and incubated for additional 4 hours at 37°C. Cells were then dried at room temperature for 15 minutes, fixed, and the DNA was denatured in order to make the incorporated BrdU more accessible for detection by the antibody. The monoclonal anti-BrdU peroxidase conjugated antibody was added to the cultures and incubated for 90 min at room temperature. After three washing steps the bound peroxidase was detected by subsequent substrate reaction. This reaction was stopped by adding 1 M H_2_SO_4_ and quantified by measuring the optical density (OD) of the yellow reaction product at a wavelength of 450nm and a reference wavelength of 655 nm using an ELISA reader. The experiments were repeated four times (four technical replicates) using CSF cultures of three different donors/patients for each condition.

### Viability assay

Cell viability (CV) was evaluated by the Alamar Blue (AB) assay (Invitrogen, Dreieich, Germany), which quantifies the reducing power of the cells. AB was added directly into culture media at a final concentration of 10% and the plate was returned to the incubator at 37°C with 95% air/5% CO_2_. Viability was measured when the medium in control wells turned from blue to pink, typically 3h after adding AB. Optical density of the plate was measured at 540 and 630 nm with a standard spectrophotometer. As a negative control, AB was added to medium without cells. The experiments were repeated four times (four technical replicates) using CSF cultures of three different donors/patients for each condition.

### Cell treatment with prolactin in vitro

Prolactin (R&D Systems / Bio-Techne, Wiesbaden, Germany) was reconstituted at 100μg/ml in sterile 4 mM HCl containing 1 mg/ml bovine serum albumin, divided into aliquots, kept frozen, and thawed only once on the day of use. The doses of PRL used in this experiment were 1000μg/ml, 100μg/ml, 10μg/ml, 1μg/ml and 0.1μg/ml, respectively.

### Measurement of IL-1β, IL-6, IL-8, FGFb, HGF, TGFβ1, EGF, MIP1α, MMP-9 and TNFα

5 hours and 24 hours after the hormone treatment, the concentration of IL-1β, IL-6, IL-8, FGFb, HGF, TGFβ1, EGF, MIP1α, MMP-9 and TNFα in each well was measured by taking a 100 μL aliquot of the supernatant of the wells. Measurements were performed by ELISA (KOMABIOTECH, Seoul, Korea) with the following measurement ranges: EGF: 250–7.8 pg/mL, FGFb: 1000-8pg/mL, HGF: 8000–60 pg/mL, IL-6: 600–10 pg/mL TGF β1: 2000–16 pg/mL, IL-1β: 1000–8 pg/mL, MMP9: 2000–16 pg/mL, TNFα: 1000–8 pg/mL, MIP1α: 500–4 pg/mL. Measured concentrations below the above values were considered as zero. The cytokine concentrations were quantified by using a human recombinant IL-1β, IL-6, IL-8, FGFb, HGF, TGFβ1, EGF, MIP1α, MMP-9 and TNFα as standard. The measurements were performed exactly following the manufactures’ ELISA-protocols. The absorbance was measured at 450 nm (Model 550 Bio-Rad Laboratories GmbH, München, Germany). The experiments were repeated four times (four technical replicates) using CSF cultures of three different donors/patients for each condition.

### Protein measurement

After taking the supernatant for ELISA, the total protein concentration of each well was measured following detachment of the cells with 150μl CelLytic^™^ M (Sigma, Deisenhofen, Germany). Protein quantity was determined according to the method of Bradford, which is based on the formation of a complex between the dye, Brilliant blue G and proteins in solution. The absorbance was measured at 595 nm and the concentrations were quantified using bovine serum albumin (BSA) as standard protein.

### Statistical analysis

GraphPad prism (GraphPad Software, San Diego, CA) and Microsoft Excel (Microsoft Corporation, Redmond, WA) were used for statistical analysis. Data are represented as mean ± standard deviation (SD). No data points were excluded. Data were assessed for Gaussian normal distribution by the D’Agostino & Pearson test and Shapiro-Wilk test. If continuous data were normally distributed Paired t test was performed for group to group comparisons. If continuous data were not normally distributed Wilcoxon matched-pairs signed rank test was performed for group to group comparisons. For multiple comparison one-way ANOVA with Tukey’s multiple comparisons test was used, if continuous data were normally distributed and Kruskal-Wallis test was used, if continuous data were not normally distributed. (ns P > 0.05; * P ≤ 0.05; ** P ≤ 0.01; *** P ≤ 0.001; **** P ≤ 0.0001)

## Results

**Fig 1:**
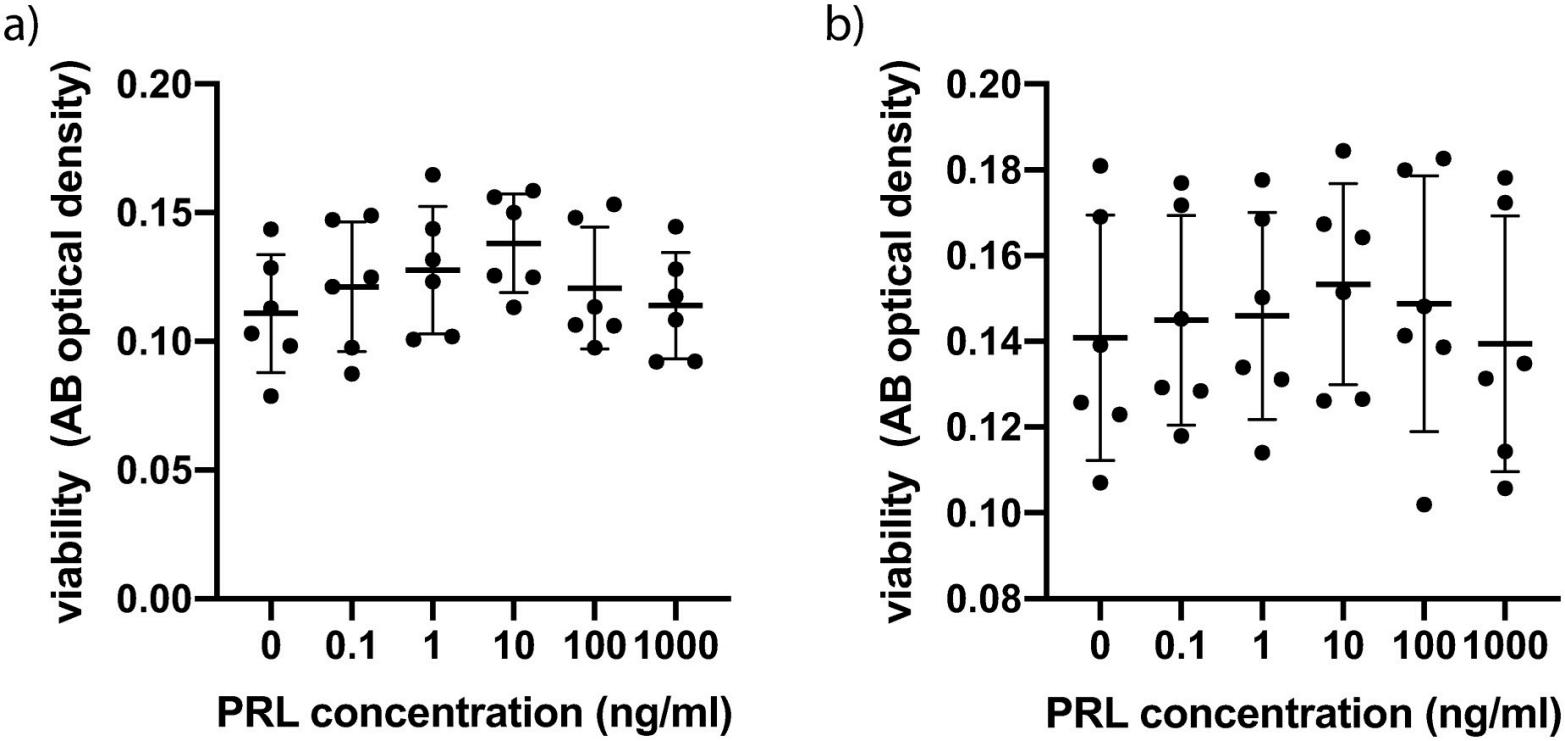
Viability of CSFs 24h after prolactin treatment. (a) Pooled male and female normal CSFs (cells from 6 individual corneas were obtained and the assay was performed on 4 separate cell-wells per individual cornea, for which the values were averaged: n = 6; 4 technical replicates each) (b) Pooled male and female KC CSFs (n = 6; 4 technical replicates each). Data plotted as mean ± SD.

Cultured CSFs were exposed to PRL at different concentrations. A concentration of 10ng/ml represents physiologic PRL blood levels, whereas 1000 ng/ml represents PRL blood levels in the third trimester of pregnancy. Cells of both 3 normal male and 3 healthy female probands as well as of 3 male and 3 female KC patients were examined. Normal CSFs exhibited the highest viability at a PRL concentration of 10ng/ml (Fig 1a). However, when pooling male and female values (n = 6) there was no significant intergroup difference employing one-way ANOVA (P = 0.4) (Fig 1a). Pooled values for male and female KC CSFs exhibited greater standard deviations within groups (Fig 1b) and also no significant intergroup difference (P>0.9). Comparing the viability means of all normal CSFs to the viability means of all KC CSFs showed no significant difference (P = 0.1, unpaired t-test) (S2 Table) and also comparing the means of all male CSFs to the means of all female CSFs yielded no significant difference (P = 0.8, unpaired t-test) (S2 Table).

Data from the BrdU proliferation assay showed a 10-fold difference between CSFs of different normal donors (S3 Table) yielding a not normally distributed dataset. An intergroup comparison with Kruskal-Wallis test showed no significant difference in proliferation between different PRL levels (P>0.9) (S3 Table). Data for KC CSFs was normally distributed but also exhibited no significant difference in proliferation between different PRL levels (P = 0.8, one-way ANOVA) (S3 Table). Further, in KC CSFs there was no significant difference in proliferation between male and female CSFs (P = 0.4, unpaired t-test) (S4 Table).

**Fig 2:**
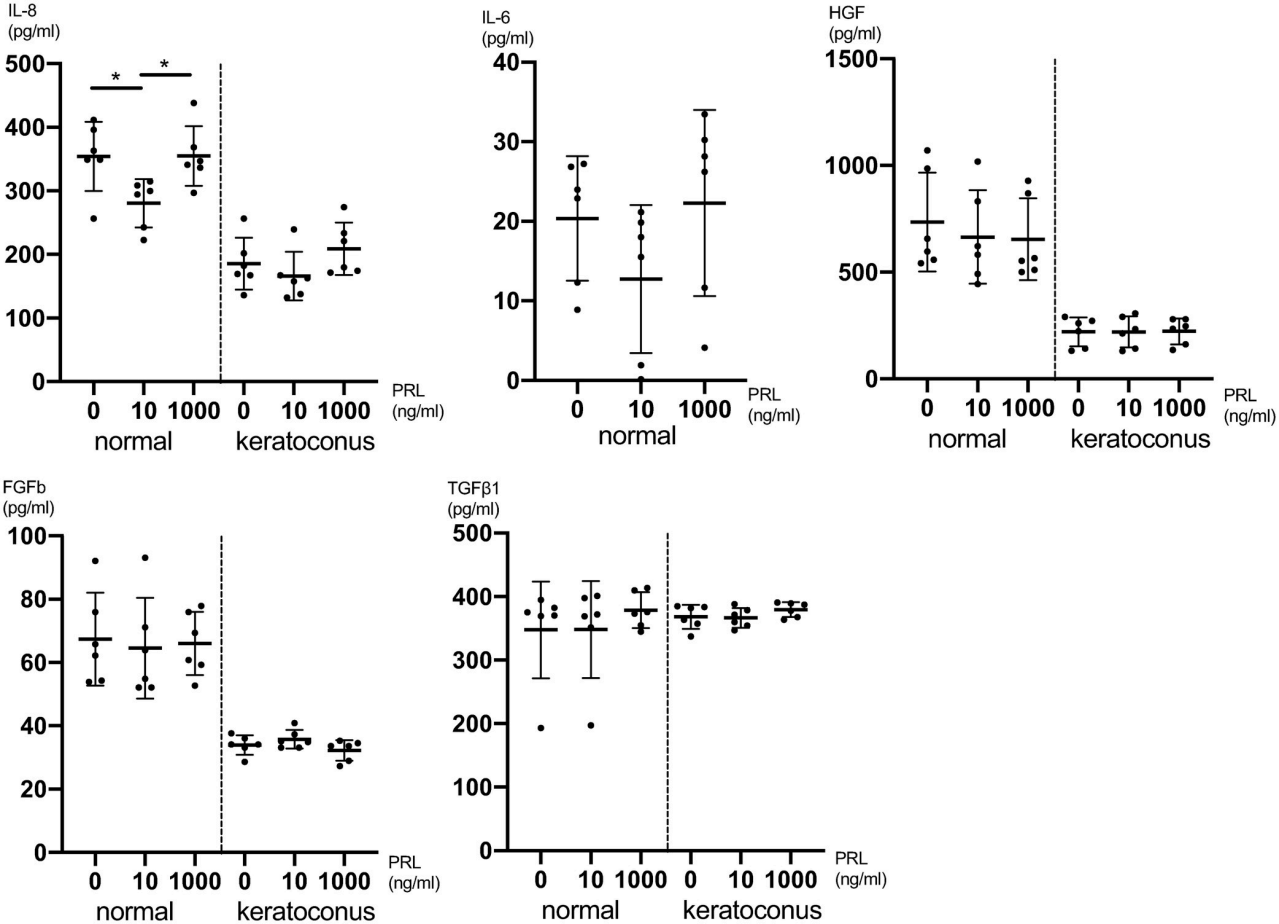
Cytokine secretion of CSFs 5h after prolactin treatment. Pooled male and female CSFs for both normal and KC (cells from 6 individual corneas were obtained and the assay was performed on 4 separate cell-wells per individual cornea, for which the values were averaged: n = 6; 4 technical replicates each). The secretion of IL-6 in KC CSFs was below the detection limit. Data plotted as individual data points, mean ± SD. (* P ≤ 0.05).

Next, we investigated the secretion of cytokines in CSFs upon PRL treatment. The concentrations of IL-8 five hours after PRL treatment were significantly higher at both 0ng/ml (P = 0.04) and 1000ng/ml (P = 0.04) compared to 10ng/ml PRL treated cells (one-way ANOVA: P = 0.02 and Tukey’s multiple comparisons test) (Fig 2). For IL-6, HGF, FGFb and TGFβ1 there was no significant difference in secretion in intergroup comparisons employing one-way ANOVA (Fig 2 and S5 Table). The secretion of IL-1β, EGF, MIP1α, protease MMP-9, VEGF, NGF, KGF and TNFα was below the detection limit in the treated and untreated cell cultures five hours after treatment.

**Fig 3:**
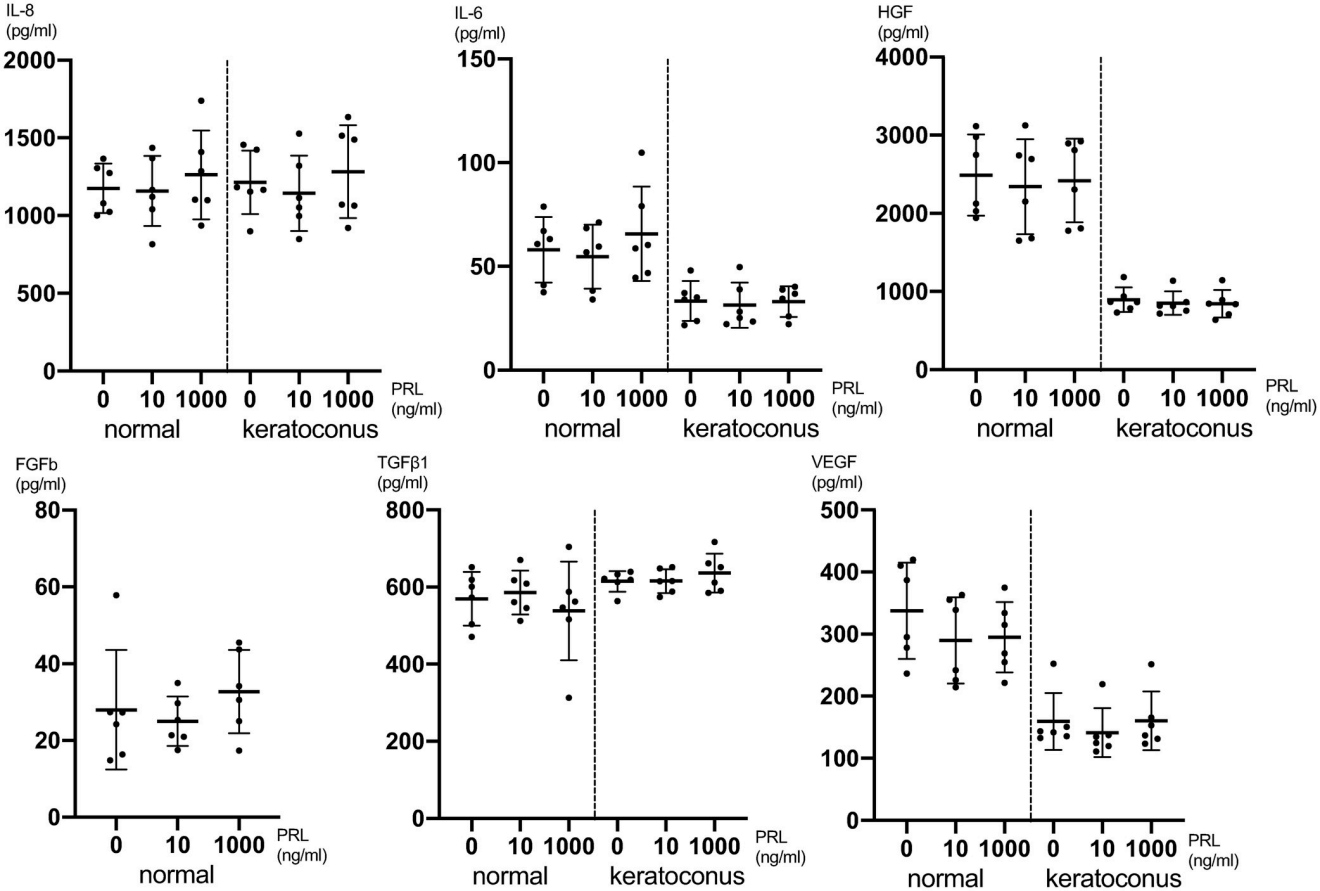
Cytokine secretion of CSFs 24h after prolactin treatment. Pooled male and female CSFs for both normal and KC (cells from 6 individual corneas were obtained and the assay was performed on 4 separate cell-wells per individual cornea, for which the values were averaged: n = 6; 4 technical replicates each). The secretion of FGFb in KC CSFs was below the detection limit. Data plotted as individual data points, mean ± SD.

24 hours after PRL treatment there was no significant cytokine expression difference detectable between different PRL concentrations for IL-8, IL-6, HGF, FGFb, TGFβ1 and VEGF employing one-way ANOVA (Fig 3 and S6 Table). The secretion of IL-1β, EGF, MIP1α, protease MMP-9, NGF, KGF and TNFα was below the detection limit in the treated and untreated cell cultures 24 hours after treatment. In general, the secretion of certain cytokines was up to 3-fold lower in KC CSFs compared to normal CSFs as can be seen in Figs 2 and 3.

**Fig 4:**
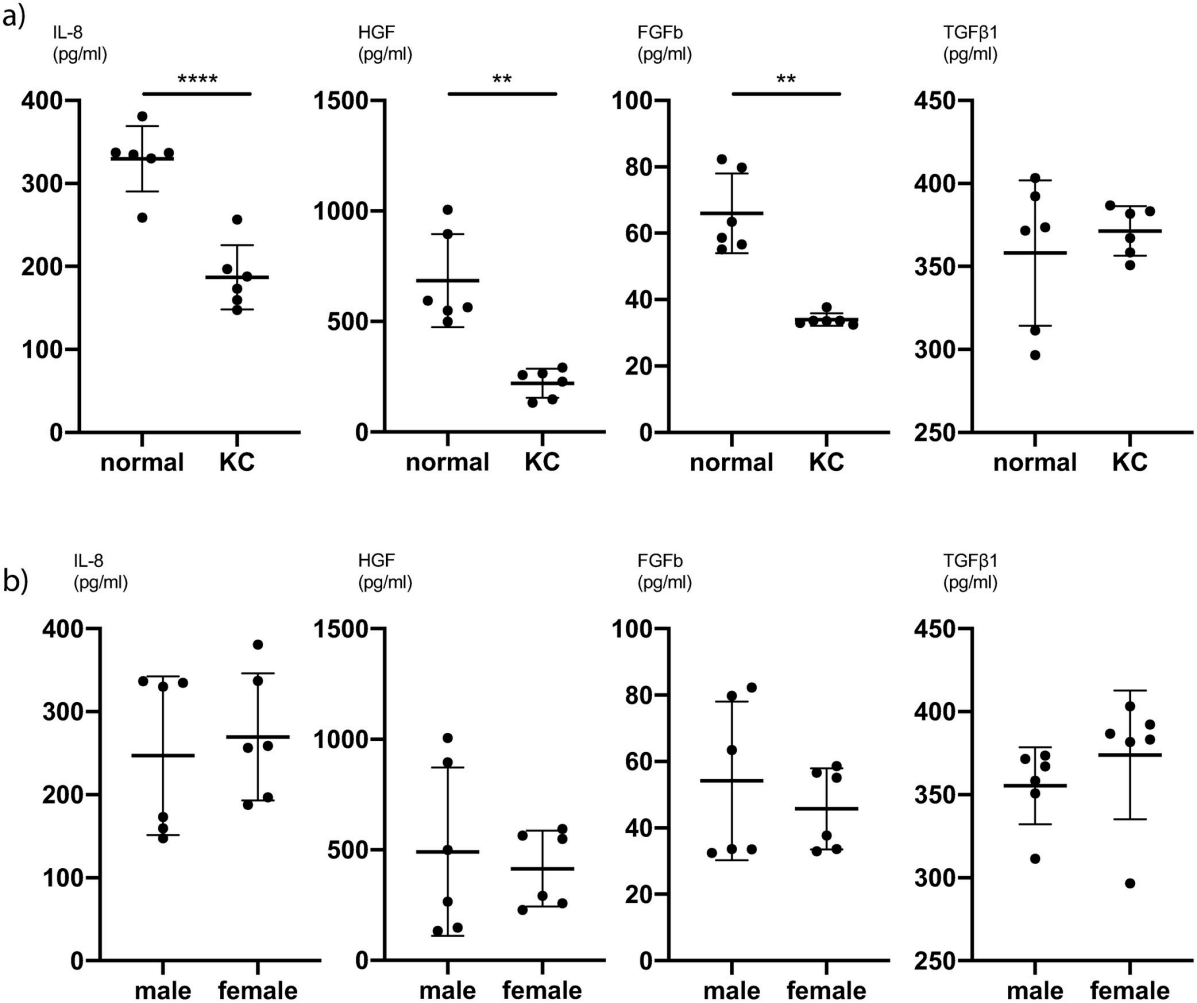
Pooled comparisons for cytokine secretion of CSFs 5h after prolactin treatment. (a) Mean values of 0ng/ml, 10ng/ml and 1000ng/ml PRL conditions, with pooled values of male and female CSFs, comparing normal and KC CSF cytokine secretion (n = 6). (b) Mean values of 0ng/ml, 10ng/ml and 1000ng/ml PRL conditions, with pooled values of normal and KC CSFs, comparing male and female CSF cytokine secretion (n = 6). Data plotted as individual data points, mean ± SD. (* P ≤ 0.05; ** P ≤ 0.01; *** P ≤ 0.001; **** P ≤ 0.0001). Calculations for reference in S7 Table.

**Fig 5:**
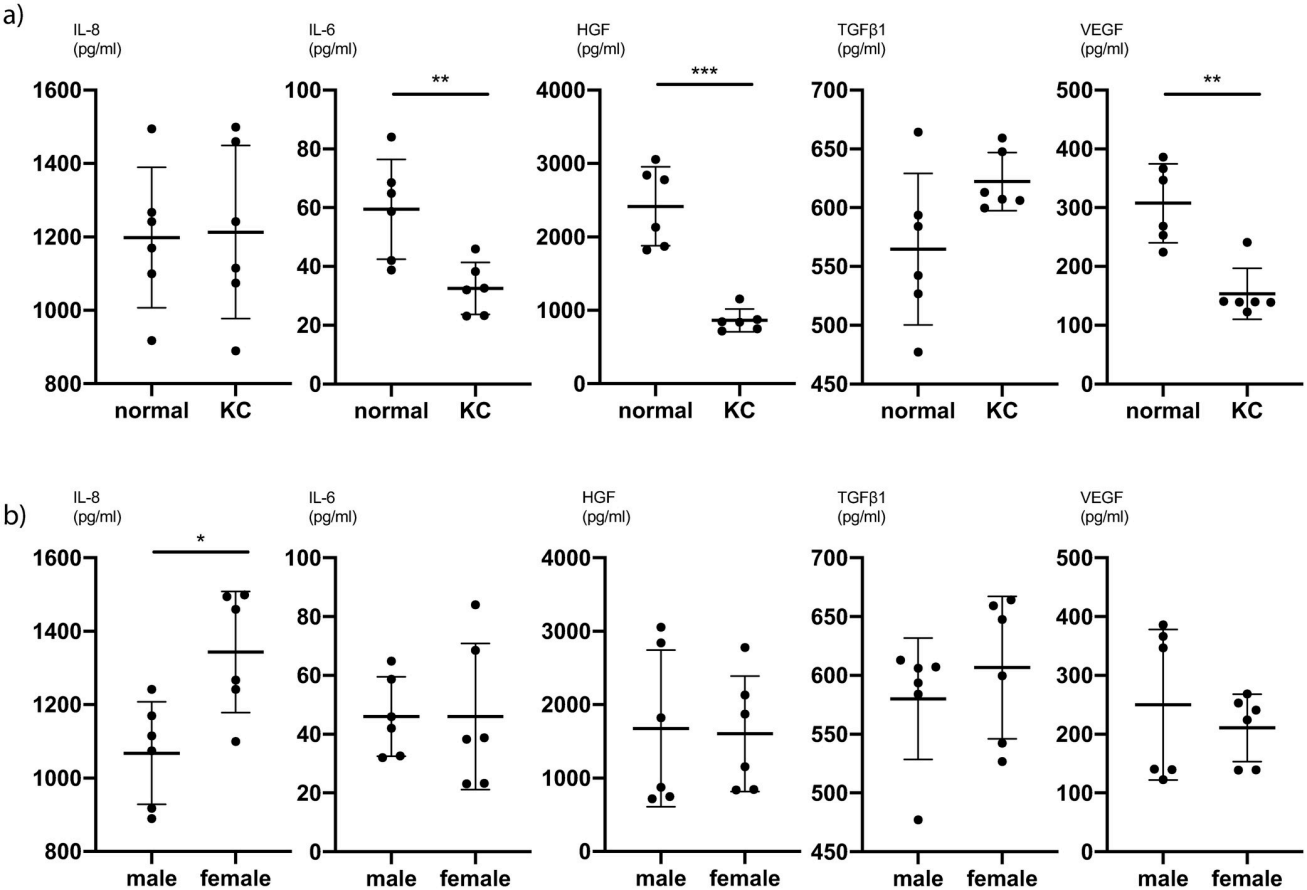
Pooled comparisons for cytokine secretion of CSFs 24h after prolactin treatment. (a) Mean values of 0ng/ml, 10ng/ml and 1000ng/ml PRL conditions, with pooled values of male and female CSFs, comparing normal and KC CSF cytokine secretion (n = 6). (b) Mean values of 0ng/ml, 10ng/ml and 1000ng/ml PRL conditions, with pooled values of normal and KC CSFs, comparing male and female CSF cytokine secretion (n = 6). Data plotted as individual data points, mean ± SD. (* P ≤ 0.05; ** P ≤ 0.01; *** P ≤ 0.001; **** P ≤ 0.0001). Calculations for reference in S8 Table.

To further analyse the difference between KC CSFs and normal CSFs, we averaged all values for different PRL exposures and compared the cytokine expression of KC CSFs and normal CSFs. We determined, that for IL-8 (P<0.0001, unpaired t-test), HGF (P = 0.002, Mann-Whitney-test) and FGFb (P = 0.002, Mann-Whitney-test) there is significantly lower cytokine secretion in KC CSFs compared to normal CSFs at our first timepoint of measurement (5 hours post PRL application) (Fig 4a and S7 Table). This trend also holds true comparing KC CSFs and normal CSFs at individual PRL concentrations (S5 Table). Further, at our second timepoint of measurement (24 hours post PRL application) there is a significantly lower cytokine secretion in KC CSFs compared to normal CSFs for IL-6 (P = 0.01, unpaired t-test), HGF (P = 0.0006, unpaired t-test), and VEGF (P = 0.004, Mann-Whitney-test) (Fig 5a and S8 Table). This trend also holds true comparing KC CSFs and normal CSFs at individual PRL concentrations (S6 Table).

We subsequently pooled the averaged cytokine expressions across PRL concentrations for KC and normal CSFs in order to compare male and female cytokine expression. Acknowledging that these datasets are wide-spread, since we observed significant differences between KC CSF and normal CSF cytokine expression, we recognize that this analysis is not very sensitive for significant differences between sexes. Still, we pooled KC CSF and normal CSF values in order to increase the number of biological replicates (n = 6) per analysis, knowing that some differences might not be detected as significant.

There was a significantly higher IL-8 secretion in female CSFs compared to male CSFs at the second timepoint of measurement (P = 0.01, unpaired t-test) (Fig 5b and S8 Table). For all other cytokines we did not find significant secretion differences between male and female CSFs (Figs 4b and 5b, S7 and S8 Tables).

## Discussion

There is increased evidence, that inflammatory pathways are involved in the pathogenesis of keratoconus (KC) [3, 18–20], although KC has traditionally been considered to be a non-inflammatory disease. Moreover, hormonal influences, particularly related to thyroid hormones, have been described to play a role in the development of KC [7, 9, 18, 21]. Interestingly, pregnancy has been associated with progression of KC or even development of acute KC, suggesting that hormonal changes occurring during pregnancy contribute to the pathogenesis of KC as well [22–25]. Prolactin (PRL) is a key pregnancy hormone, whose effect on corneal physiology is yet to be investigated, and therefore we focused on this molecule.

With respect to viability and proliferation our experiments did not show significant differences between CSFs exposed to different PRL concentrations. One has to acknowledge though, that normal CSFs exhibited a dose dependent peak at 10ng/ml (Fig 1a), even though the differences compared to other PRL concentrations were not statistically significant. Follow up studies with n>6 might show a significantly higher viability of CSFs exposed to 10ng/ml PRL, compared to other PRL concentrations. Further our data show a significantly lower IL-8 concentration in normal CSFs exposed to 10ng/ml PRL compared to 0ng/ml and 1000ng/ml at 5 hours post exposition (Fig 2). This tendency of lower cytokine secretion at the near physiologic 10ng/ml PRL exposure holds true for various other cytokines (Figs 2 and 3), albeit the differences are not statistically significant (IL-6, VEGF, FGF, FGFb). At the same time our data do not provide a greater PRL exposure dependent effect on KC CSFs compared to normal CSFs as assessed by cytokine secretion. Recognizing that only for IL-8 in normal CSFs there was a significant PRL dependent difference and that a study with greater number of donor corneas has to be conducted to verify this trend, our data in part provide a link between PRL exposure and altered cytokine secretion. As all of the examined cytokines have been characterized as contributors to KC development [26, 27], our data provide a possible link between altered stromal PRL concentrations and KC progression. This is in line with findings of Stachon et al., who described decreased aqueous humor PRL concentrations in patients with KC [14]. Our results can also be put in context with findings of Sharif et al., who reported that prolactin-induced protein (PIP) is downregulated in tears, plasma and saliva of patients with KC when compared to healthy controls [28]. Consequently PIP has been proposed as a KC biomarker. PIP expression itself is regulated by androgens, estrogens and PRL [29].

Several studies have examined the role of cytokines in the context of KC [3, 12, 13, 18, 20, 30]. Pahuja et al. described elevated levels of MMP-9, TNF-a, and IL-6 but reduced IL-10 and tissue inhibitor of metalloproteinases 1 (TIMP-1) in epithelial cells collected from the cone apex of Bowman’s layer breached KC patients [12]. Ionescu et al. reported significantly elevated IFN gamma, IL-10, IL-1 beta, IL-4, IL-6 and TNF-a concentrations in the tear fluid of KC patients [20]. Our data show significantly lower secretion of IL-8, HGF and FGFb in KC CSFs compared to normal CSFs 5 hours after exposure to PRL (Fig 4a). Likewise, we can report significantly lower secretion of IL-6, HGF and VEGF 24 hours after exposure to PRL as determined by cytokine ELISA (Fig 5a). Other groups have described both significantly higher and significantly lower levels of certain cytokines in KC anterior eye segments compared to controls [12, 18]. Our results contribute to these existing findings, with an emphasis on significantly lower cytokine secretion in KC CSFs compared to normal controls. Finally, our cytokine ELISA exhibited significantly higher IL-8 levels in female CSFs compared to male CSFs 24 hours after exposure to PRL (Fig 5b). Since all other cytokine ELISAs showed no significant difference between male and female CSFs, we would not easily infer a distinct response of female CSFs towards PRL exposure.

## Supporting information

S1 Table Viability of CSFs 24h after PRL treatment. (XLSX)

S2 Table Viability of CSFs 24h after PRL treatment—Grouped comparisons. (XLSX)

S3 Table Proliferation of CSFs 24h after PRL treatment. (XLSX)

S4 Table Proliferation of CSFs 24h after PRL treatment—Grouped comparisons. (XLSX)

S5 Table Cytokine secretion of CSFs 5h after PRL treatment. (XLSX)

S6 Table Cytokine secretion of CSFs 24h after PRL treatment. (XLSX)

S7 Table Cytokine secretion of CSFs 5h after PRL treatment—Grouped comparisons. (XLSX)

S8 Table Cytokine secretion of CSFs 24h after PRL treatment—Grouped comparisons. (XLSX)
